# A Textbook of Legal Medicine and Toxicology

**Published:** 1903-09

**Authors:** 


A Textbook of Legal Medicine and Toxicology. Edited by Frederick Peterson, M. D., Chief of Clinic, Nervous Department of the College of Physicians and Surgeons, New York; and Walter S. Haines, M. D., Professor of Chemistry, Pharmacy, and Toxicology, Rush Medical College. Volume I. Philadelphia, New York, London: W. B. Saunders & Company. 1903. (Per volume: cloth, $5.00 net; sheep or half-morocco, $6,00 net.)

This book is intermediate between the small manuals and the encyclopedias. The authors have wisely considered the masses and the needs of the profession at large in their choice of weights and measures. They have adhered to the English system, as this is by far the most easily understood by juries.

The contributors to this volume, beside the editors, are Samuel Treat Armstrong, Pearce Bailey, Lewis Balch, James Ewing, Graeme M. Hammond, Smith Ely Jelliffe, all of New York; John

Chalmers DaCosta, Philadelphia; J. T. Eskridge, late of Denver; Josiah N. Hall and Edward Jackson, Denver; Ludwig Hektoen, Chicago; Charles Gilbert Chaddock, Saint Louis; F. W. Langdon, Cincinnati, and Allen J. Smith, Galveston.

The subjects considered are: expert evidence, the technic of medico-legal postmortem examination, identity, signs of death, sudden death, death from cold, heat and starvation, asphyxia, lightning and electricity, wounds, burns and scalds, destruction and attempted destruction of the human body by fire and chemicals, railway injuries, medical jurisprudence of life insurance and of accident insurance, medico-legal aspects of vision and audition, speech disorders, inebriety, the stigmata of degeneration, insanity, idiocy, imbecility and feeble mindedness, and mental perversions of the sexual instinct. Each subject is fully treated and numerous cases are quoted, which illustrate the technical points in expert testimony. Thus Dr. Haines in his article on destruction of the human body by fire and chemicals cites two famous cases: the Professor Webster case and the Leutgert case. The directions to experts are summarised in a few rules, which, if followed, will materially assist a witness in many difficult cases.

The book is profusely illustrated, many of the plates being colored so as to show changes by burns, and asphyxia from hanging. We believe the greatest usefulness of such a book is that it affords a rapid means of reference to authorities for any special case. In our experience the great need an expert experiences during a trial is a book to which he can quickly refer upon any medical point in dispute. No matter how well qualified or experienced a man may be, the occasion every now and then arises when a reliable book which quotes well-known authoritative cases, is a necessity. A good index at such a time adds much to the value of the book. We believe that this book will be a popular one with lawyers and physicians. J. W. P.

